# Retraction: Spatial distribution of elements, environmental effects, and economic potential of waste from the Aksu ferroalloy plant [Kazakhstan]

**DOI:** 10.1371/journal.pone.0309086

**Published:** 2024-08-15

**Authors:** The PLOS ONE Editors

After this article [1] was published, the following concerns were raised:

- The article [1] contains citations, including by the last author, that do not support the statements in which they are cited. The authors did not respond to address this concern, and as this issue remains unresolved the reliability of information reported in the article is in question.
- There are concerns about authorship, competing interests, and peer review.
- A member of the *PLOS ONE* Editorial Board reviewed the article [1] and stated that based on the reported methodology the study appears to be based on single point data with no replicates collected during sampling. This has implications for the data quality and the reliability of the article’s results and conclusions.

The *PLOS ONE* Editors retract this article [1] due to the above concerns. We regret that the issues were not addressed prior to the article’s publication.

LDD responded but expressed neither agreement nor disagreement with the editorial decision. RS, ZB, RU, YN, ZS, ZB, AK, IV, AB, and RND either did not respond directly or could not be reached.
